# Variants in the L12 linker domain of KRT10 are causal to atypical epidermolytic ichthyosis

**DOI:** 10.1111/1346-8138.17395

**Published:** 2024-07-29

**Authors:** J. J. A. J. van der Velden, M. W. van Gisbergen, M. A. F. Kamps, R. Janssen, G. F. H. Diercks, P. M. Steijlen, M. van Geel, M. C. Bolling

**Affiliations:** ^1^ Department of Dermatology Maastricht University Medical Centre+ Maastricht The Netherlands; ^2^ GROW‐School for Oncology and Reproduction Maastricht University Maastricht The Netherlands; ^3^ Department of Dermatology, UMCG Center of Expertise for Blistering Diseases University Medical Center Groningen, University of Groningen Groningen The Netherlands; ^4^ Department of Pathology, UMCG Center of Expertise for Blistering Diseases University Medical Center Groningen, University of Groningen Groningen The Netherlands; ^5^ Department of Clinical Genetics Maastricht University Medical Centre+ Maastricht The Netherlands

**Keywords:** epidermolytic ichthyosis, keratin 10, KRT10, linker domain, peeling skin

## Abstract

Epidermolytic ichthyosis (EI) is a type of congenital ichthyosis, characterized by erythema and blistering at birth followed by hyperkeratosis. EI is caused by pathogenic variants in the genes *KRT1* and *KRT10*, encoding the proteins keratin 1 (KRT1) and keratin 10 (KRT10), respectively, and is primarily transmitted by autosomal‐dominant inheritance, although recessive inheritance caused by nonsense variants in *KRT10* is also described. The keratins form a network of intermediate filaments and are a structural component of the cytoskeleton, giving strength and resilience to the skin. We present three cases of mild EI caused by pathogenic KRT10 variations in the L12 linker domain. To our knowledge, this is the first time L12 linker domain pathogenic variants are identified in *KRT10* for EI. The aim of this study was to identify gene variants for patients with EI in *KRT1* or *KRT10*. To establish the pathogenicity of the found variations in *KRT10*, we evaluated all patients and available family members clinically. Genetic analyses were performed using Sanger sequencing. Vectors containing wild‐type or mutated forms of *KRT10* were transfected into HaCaT cells and analyzed by high‐resolution confocal microscopy. Genetic analysis of *KRT10* identified a heterozygous de novo variant c.910G>A p.(Val304Met) in family 1, a familial heterozygous variant c.911T>C p.(Val304Ala) in family 2, and a familial heterozygous variant c.917T>C p.(Met306Thr) in family 3. All identified missense variants were located in the L12 linker domain of KRT10. In vitro study of aggregate formation of the missense variants in KRT10 only showed a very mild and not quantifiable aggregate formation in the KRT10 network, compared with the wild‐type sequence. We report three different novel missense variants in the L12 linker domain of KRT10 in patients with an atypical, milder form of EI resembling peeling skin syndrome.

## 1 INTRODUCTION

Keratins form a network of intermediate filaments and are an abundant structural component of the cytoskeleton, giving strength and resilience to the skin and protecting it from being damaged by physical stress such as friction.^1^ Epidermolytic ichthyosis (EI) is a type of congenital ichthyosis, characterized by erythema and blistering at birth followed by hyperkeratosis of varying severity and extensiveness.^2^, ^3^ EI is caused by pathogenic variants in either *KRT1* encoding keratin 1 or *KRT10* encoding keratin 10 and is primarily transmitted by autosomal dominant inheritance, although recessive inheritance is also observed in EI caused by nonsense variants in *KRT10*.^4^, ^5^ Subsequently, the network becomes less stable and therefore more susceptible to stress such as friction and heat, causing disruption of the filaments. Hence, upon external stress conditions, these keratin filaments can aggregate resulting in loss of cellular adhesion and acantholysis. Clinically, this presents itself as blisters and erosions in patients.^6^, ^7^ The blisters that are observed in EI become less frequent later in life, while hyperkeratosis becomes more pronounced. In addition, the increased proliferation of the suprabasal keratinocytes to compensate for the compromised skin barrier results in ichthyosiform lesions in these patients.^7^, ^8^

KRT10, is a type I keratin expressed in the spinous and granular cell layer of the epidermis and is a binding partner to KRT1.^9^, ^10^, ^11^ Keratins are found to form heterodimer consisting of one type I and one type II keratin protein, and these heterodimers bind in an antiparallel manner to form a tetradimer, by a pocket/knob mechanism.^10^, ^12^ Only type II keratins are found to have a pocket/knob–pocket interaction and therefore contribute to the formation of the tetradimer, and the stability is mainly dependent on the KRT1 in the heterodimer of KRT10/KRT1. In addition to KRT10, KRT1 can also heterodimerize with KRT9, exclusively in the palmoplantar skin where KRT9 is expressed.^13^

KRT1 and KRT10 heterodimerize through their rod domains. These rod domains are α‐helical segments that are separated by nonhelical linker domains (L1, L12, and L2).^14^ When keratins 10 or 1 are defective, the keratin network becomes instable, leading to keratin clumping, clinically resulting in EI.^5^ For *KRT1* pathogenic variants, a genotype–phenotype correlation has been reported in EI. In general, the less severe phenotypes occur with variants in the α‐helical regions and the L12 linker domain, whereas the more severe phenotypes manifest in patients harboring variants in the helix boundary motifs.^15^ For the type II keratin 1, L12 linker domain pathogenic variants have been reported in atypical bullous congenital ichthyosiform erythroderma of Brocq. Superficial blisters, erosions, and erythroderma at birth are reported and hyperkeratosis is observed on the palmoplantar skin when patients get older, while the other symptoms diminish.^14^, ^15^, ^16^ Up to now, almost exclusively pathogenic variants in *KRT10* have been reported to occur in the highly conserved α‐helical boundary domains for EI.

Herein, two cases of EI and one patient initially diagnosed with peeling skin syndrome are presented. In all three cases, pathogenic KRT10 variations in the L12 linker domain were identified and are reported for the first time.

## 2 MATERIALS AND METHODS

### 2.1 Clinical characteristics of the patients

**FIGURE 1:**
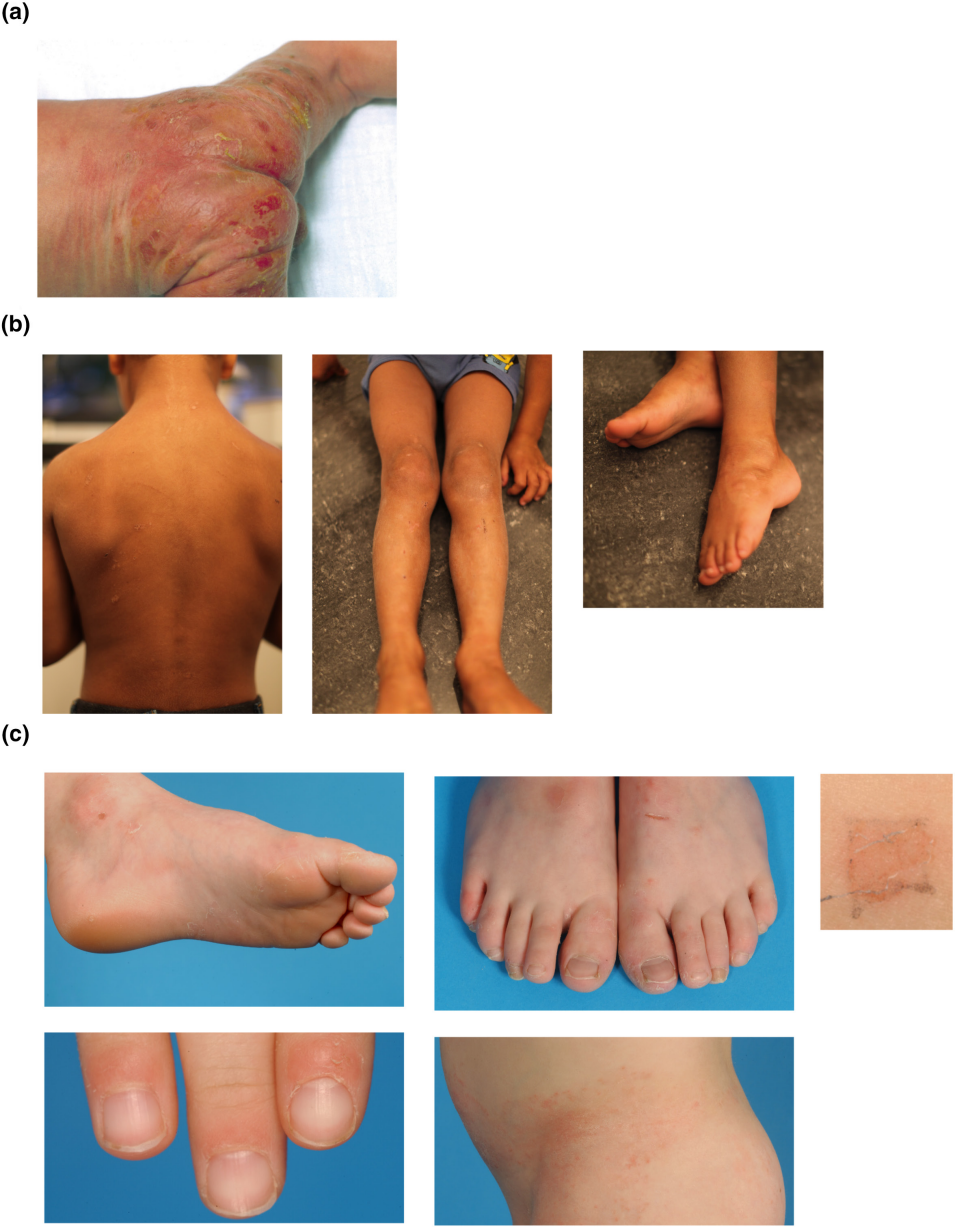
Clinical representations of the index patients of all three families. (a) Clinical presentation of the index patient of family 1, patient II‐1, 1 day after birth. Erythroderma, superficial blistering, and erosions can be observed. (b) Clinical presentation of the index patient of family 2 patient II‐1, here hyperkeratotic plaques, mildly erythematous and desquamation can be observed. (c) Clinical presentation of the index patient of family 3, patient IV‐1. Superficial desquamation with underlying mild erythema and a collarette border and erythematous plaques and papules are observed. Plantar subungual hyperkeratosis was also noted. Upon friction, desquamation was induced (top right panel).

In family 1, only one patient (II‐1) was affected with EI. The patient presented with erythroderma and superficial erosions in the neonatal period (Figure 1a). In childhood, blisters occurred upon friction, resulting in superficial erosions on the neck and hands and in skin folds (Figure S1). However, no signs of hyperkeratosis or palmoplantar keratoderma (PPK) were observed. Histology showed eosinophilic spongiosis, and immunofluorescent images revealed separation of the stratum granulosum (Figure S1). Electron microscopy (EM) analysis showed a subcorneal intercellular separation. No clear signs of keratin clumping could be observed, but keratohyalin aggregation was shown on biopsy at 2 years of age.

The index patient with EI (II‐1) of family 2 showed erythroderma and fluid‐filled blisters in the neonatal period. Later in childhood, hyperkeratotic and erythematous plaques on the trunk and extremities were observed (Figure 1b). EM analysis of a skin biopsy revealed subcorneal separation and clumping of the keratin in the stratum granulosum. The father (I‐1) had similar symptoms on his head and body as an infant. Currently, ichthyosiform plaques and superficial erosions were observed in the index patient and father (Figure S2).

The index patient (IV‐1) of family 3 clinically presented with redness and superficial desquamation of the skin at birth. He was initially diagnosed with peeling skin syndrome (Figure 1c). Blisters formed at points of friction, and signs of hyperkeratosis, as well as a few intracorneal cracks, were microscopically observed. EM showed intercellular separation of the corneocytes in the stratum corneum. Next to the globular keratohyalin granules also enlarged vacuolated dense bodies and a cytoplasmic edema in the stratum granulosum were observed. No clumping of the tonofilaments was found. Stain findings for filaggrin and KRT14 were normal, e.g. filaggrin showed positive staining for the stratum granulosum and stratum corneum, whereas KRT14 was absent at this location. Upon friction, separation of the substratum of the stratum granulosum was observed histologically/on EM. The phenotype reduced over the years and at adulthood the patient still showed a very mild plantar hyperkeratosis (Figure S3). The mother (III‐2) has similar symptoms and shows plantar hyperkeratosis and very mild peeling (Figure S3). Upon friction, subcorneal separation and edema until the midstatum spinosum was seen on EM.

### 2.2 Genetic analysis

Genetic analysis was performed using Sanger sequencing (Applied Biosystems 3730XL DNA analyzer) and evaluated in Mutation Surveyor (SoftGenetics). All coding sequences of *KRT1* and *KRT10* were analyzed (primer sequences on request).

### 2.3 High‐resolution confocal microscopy

A vector (pCMV6‐XL5‐KRT10; Origene # SC122561) containing the wild‐type sequence of *KRT10* was used to reclone *KRT10* into a pEGFP‐C1 vector in which EGFP was replaced by Citrine (kindly provided by R. Tsien). The missense variants (Arg156Cys, Val304Met, Val304Ala, and Met306Thr) were introduced by using side‐directed mutagenesis (Stratagene). Constructs containing either the wild‐type or a mutated form of *KRT10* were transfected into HaCaT (human immortalized keratinocytes) with GENIUS DNA Transfection Reagent (Westburg) and selected upon G418 (Invitrogen). Cells were cultured in Dulbecco's Modified Eagle Medium (Gibco), supplemented with 10% (v/v) fetal bovine serum (Sera Lab).

Cells were seeded on glass slides and fixated with 4% paraformaldehyde in phosphate‐buffered saline (PBS; Sigma‐Aldrich). After fixation, nuclei were stained with DAPI (4′,6‐diamidino‐2‐phenylindole; 0.5 ug/mL in PBS). Slides were enclosed using mowiol mounting medium and analyzed using a Leica TCS SP8 CARS confocal microscope with stimulated emission depletion.

## 3 RESULTS

### 3.1 Identification of missense variants in the L12 linker domain of KRT10

**FIGURE 2:**
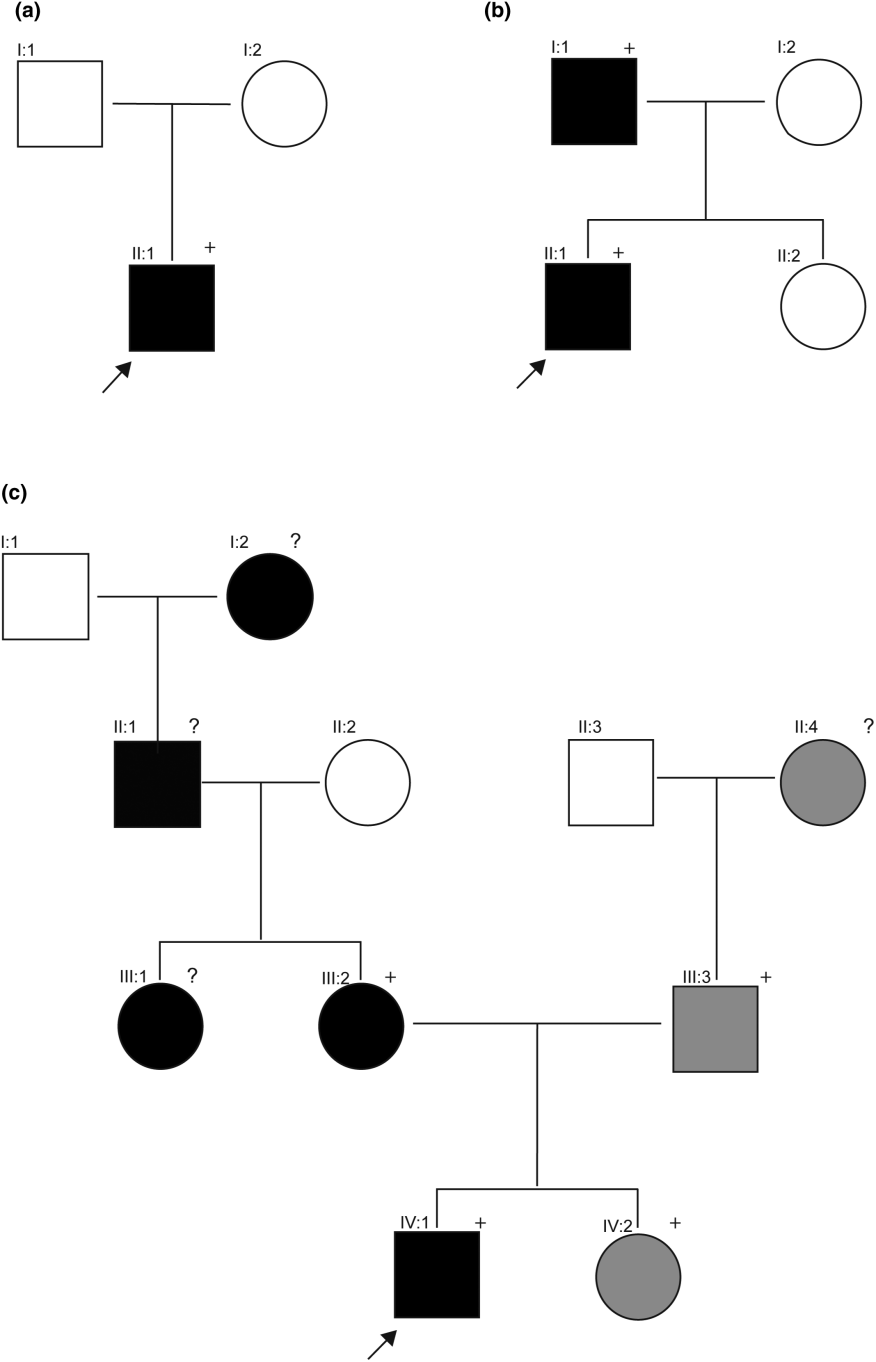
Pedigrees of the different families. (a) Pedigree of family 1, the index patient (II‐I) with heterozygous variant p.(Val304Met). (b) Pedigree of family 2, the index patient (II‐I) is heterozygous for variant p.(Val304Ala). The father (I‐I) is also heterozygous for the variant. (c) Pedigree of family 3, the index patient (IV‐1) and the family members heterozygous for the keratin (KRT) 10 p.(Met306Thr) variant (black symbols), while the family members with grey symbols have a KRT5 p.(Arg187Pro) heterozygous variant. The family members with + were genetically tested, and family members with an ? were not analyzed for the variations. The arrows indicate the index patients, the filled symbols are affected patients, the squares are males, and the circles are females.

**FIGURE 3:**
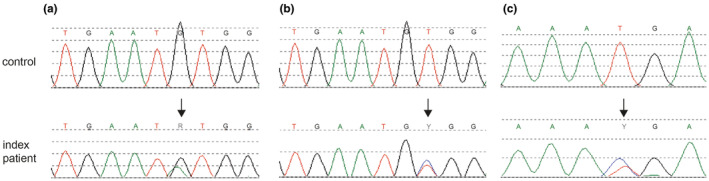
Genetic analysis of keratin (KRT) 10 in the index patients of three independent families. (a) The index patient (patient II‐1) of family 1 harbors a heterozygous c.910G>A variant in *KRT10* (arrow). (b) The index patient (patient II‐1) of family 2 harbors a heterozygous c.911T>C variant in *KRT10* (arrow). (c) The index patient (patient II‐1) of family 3 harbors a heterozygous c.917T>C variant in *KRT10* (arrow). Top panels, reference *KRT10* (NM_000421.3) control DNA sequence; lower panels, patient DNA sequence.

**FIGURE 4:**
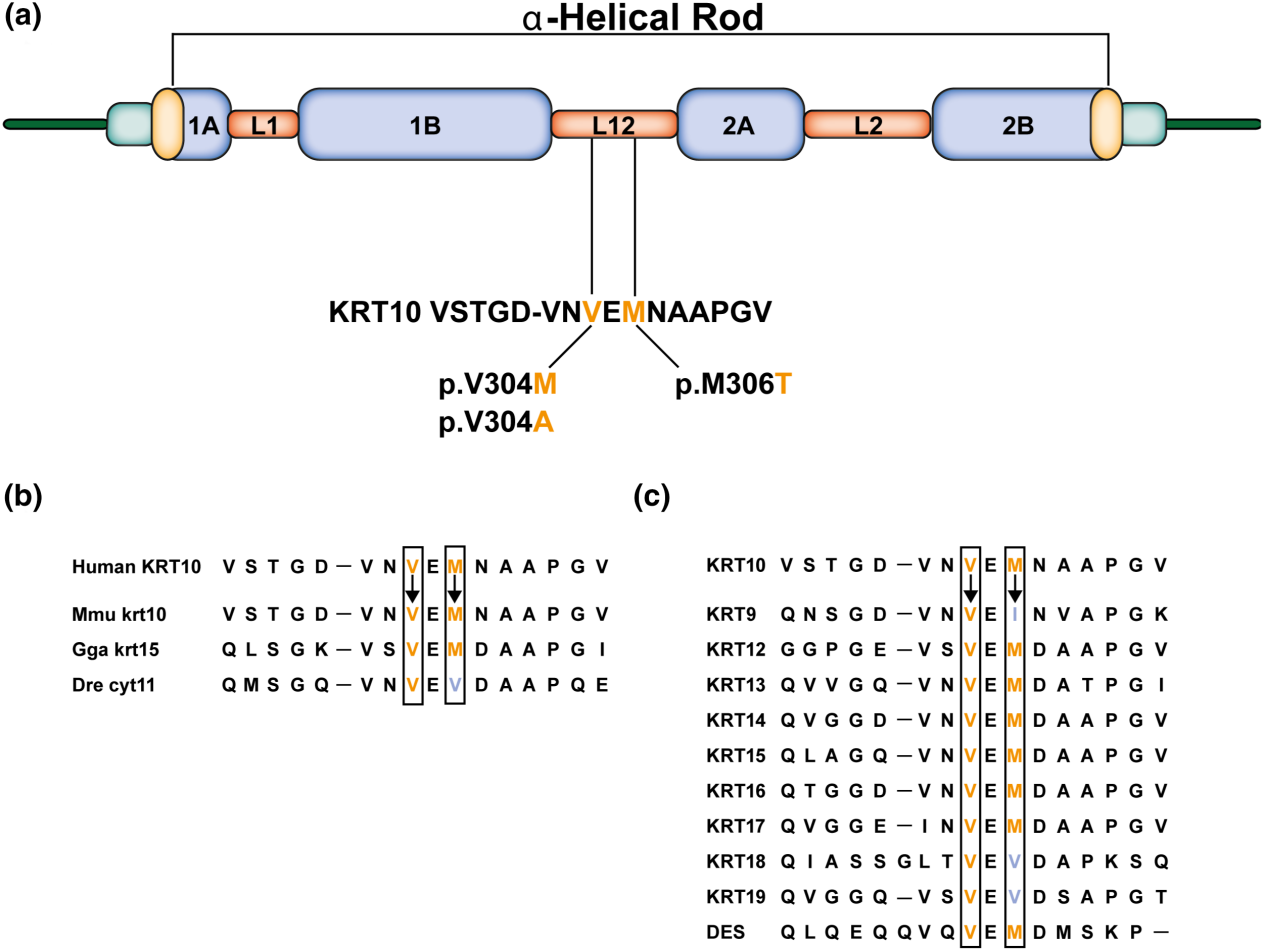
Conservation of the linker L12 protein sequence in keratin (KRT) 10 orthologues and paralogues. (a) A schematic representation that p.Val304Met, p.Val304Ala, and p.Met306Thr are located in the L12 linker domain of the α‐helical rod of KRT10. (b) The conservation between different species for the amino acids at position p.Val304 and p.Met306 in KRT10 and its orthologues (Mmu, *mus musculus*; Gga, *Gallus gallus*; Dre, *Danio rerio*). (c) Conservation of the amino acids at position p.Met304 and p.Val306 in KRT10 and their corresponding amino acids for the different keratin type I paralogues (desmin [DES]). Paralogous and orthologues of KRT10 were aligned with ClustalW.

Sequencing of *KRT10* revealed a heterozygous de novo variant c.910G>A resulting in an amino acid substitution of valine to methionine, p.(Val304Met) in family 1. The parents did not show this transition. For family 2, *KRT10* sequencing of the index patient (patient II‐I) identified a heterozygous variant c.911T>C putatively resulting in an amino acid substitution of the same valine to an alanine, p.(Val304Ala). Also, his affected father was heterozygous for this variant (patient I‐I). In the index patient of family 3 (patient IV‐1), a heterozygous *KRT10* variant, c.917T>C resulting in an amino acid substitution of methionine to threonine, p.(Met306Thr) was identified. Also, his affected mother (III‐2) had the variant (see Figure 2 for pedigrees). Noteworthy, in an independent diagnostic screening, in family members of the paternal side (III‐3) and the sister (IV‐2) of the index patient, a *KRT5* pathogenic variant c.560G>C p.(Arg187Pro) was found, establishing the diagnosis of localized epidermolysis bullosa simplex (EBS_loc_).^17^ However, none of the family members had a *KRT5* and *KRT10* variation simultaneously. Herein, all of the described *KRT10* variants occured in the L12 linker domain of KRT10 (Figure 3). The amino acid sequence of the L12 linker domain spans approximately amino acids 295–317 of KRT10^18^ and all identified missense variants are located in this region (Figure 4a).

### 3.2 Conservation of the missense variants in the L12 linker domain of KRT10

The reported variants of both index patients in families 1 and 2 resulted in amino acid changes at position 304. Valine at codon 304 is highly conserved between human type I keratin paralogues and the orthologues of *KRT10* in other species (Figure 4a,b). The variant in family 3 affecting the methionine at position 306 is likewise conserved in the L12 linker domain of other species and paralogues in humans (Figure 4b). Although the methionine at this position is sometimes found to be a valine or isoleucine, these amino acids belong to the same group with hydrophobic side chains, unlike threonine (polar uncharged) (Figure 4c).

### 3.3 In vitro study of aggregate formation of the missense variants in KRT10

**FIGURE 5:**
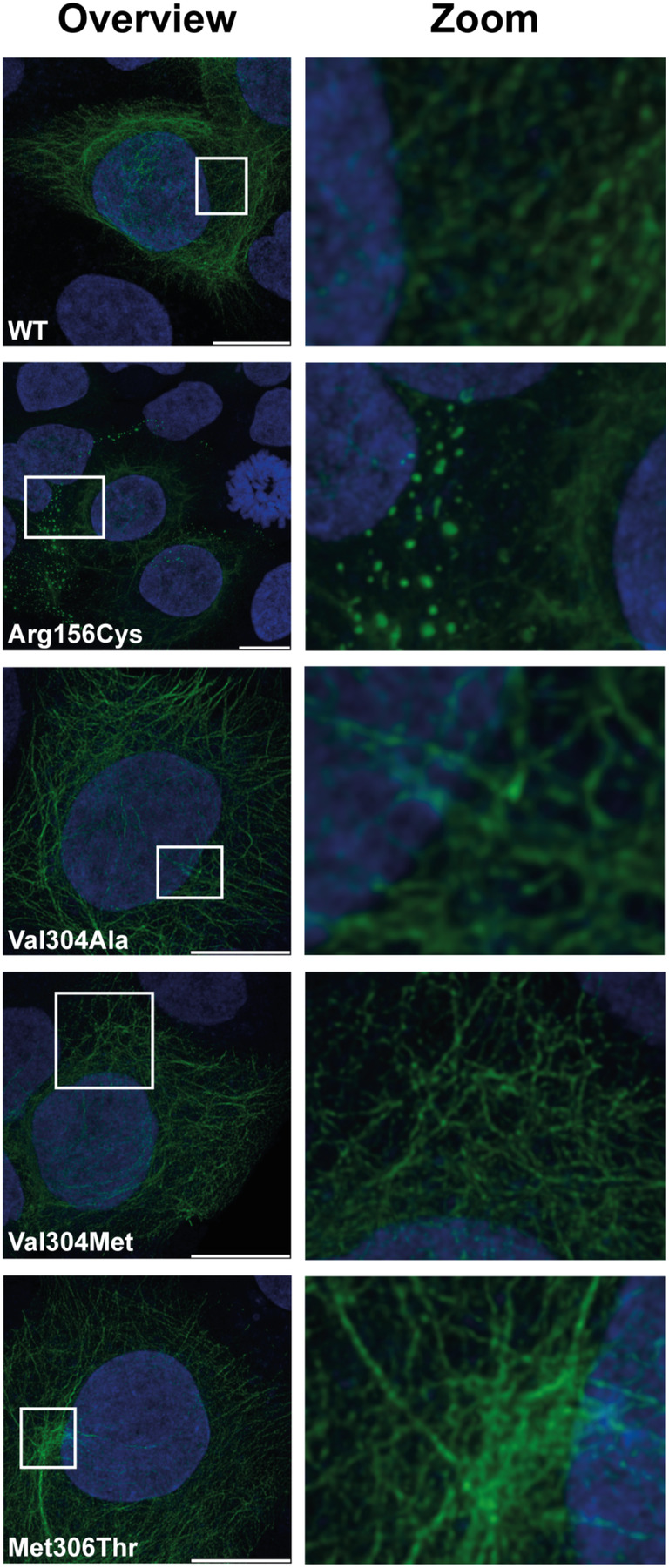
Keratin (KRT) 10 aggregate formation in transfected HaCaT cells. Stimulated emission depletion confocal images of HaCaT cells transfected with either wild‐type or a mutated KRT10 to show localization and formation of the KRT10 intermediate filament network (green). Cells transfected with Arg156Cys show aggregate formation of KRT10 and serve as a positive control. The nucleus is depicted in blue (scalebar = 10 μm).

To evaluate the influence of the identified variations on the KRT10 network in keratinocytes, transfection experiments were performed. For severe EI, a pathogenic variant in KRT10 has been reported (p.Arg156Cys).^19^ For this variant, a clear formation of KRT10 aggregates was found in keratinocytes. For the three newly identified variants in the L12 linker domain of KRT10, only a very mild and small aggregate formation in the KRT10 network was observed compared with the wild‐type sequence under normal culture conditions. However, this could not be further quantified (Figure 5). To assess whether stress evokes the aggregate formation, cells were exposed to stress conditions as previously described.^14^, ^15^ Unfortunately, the conditions for the cells exposed to osmotic shock generated no reproducible data and was therefore inconclusive.

### 3.4 Variant classifications

All variants were classified according to the American College of Medical Genetics (ACMG) standards and guidelines.^20^ None of the variants were present in control populations (Genome Aggregation Database, gnomAD v4.1.0). Variant c.910G>A was classified as likely pathogenic because of its de novo nature (PS2, PM1, PM2, PP3). Both other variants, c.911T>C and c.917T>C, were also classified as likely pathogenic, since segregation in affected family members was confirmed (PM1, PM2, PP1, PP3). Since functional studies of keratin aggregate formation was not conclusive, this was not included in the classification of the variants. However, proven pathogenic identical amino acid substitutions on equivalent positions in paralogous keratin genes (see Discussion and Conclusion) is not yet part of the ACMG classification guidelines and should be considered to be beneficial in variant classification.

## 4 DISCUSSION AND CONCLUSION

Structural domains of intermediate filaments such as KRT10 are highly conserved between species and contain a central α‐helical rod domain (subdivided into 1A, 1B, 2A, and 2B), which are separated by three short linker regions (L1, L12, and L2). Previously, L12 linker pathogenic variants have been reported for KRT14, also a type I keratin filament, and in suprabasal keratin KRT1, a type II keratin filament.^14^, ^15^, ^16^, ^17^, ^21^, ^22^, ^23^, ^24^, ^25^ Comparable to the KRT10 variants, on the equivalent position of KRT14 (p.Val270) in the L12 linker domain, causal variants were found in patients with epidermolysis bullosa simplex (EBS).^21^, ^22^, ^23^ Also, for the p.Met306Thr missense variant, a comparable substitution in the L12 linker domain for KRT14 (p.Met272Thr) has been reported in families with EBS.^24^, ^25^ The KRT10 variations described here can potentially cause a disruption of the keratin network, as has previously been shown in the L12 domain of KRT1.^14^, ^15^ For KRT14, it was suggested that the p.Val270Met substitution influences the length or flexibility of the L12 linker domain, thereby affecting the assembly of the keratin filaments.^14^, ^21^

In EBS, the position of the pathogenic variants in the L12 linker domain of KRT14 seem to correlate to a mild phenotype (EBS‐Weber‐Cockayne type or EBS‐localized), in line with the milder phenotype in our patients with L12 linker domain variants in KRT10. This would suggest that variants affecting the linker domains of KRT1 and KRT10 cause a mild phenotype and for KRT10, this is often unnoticed. In our in vitro study, only a very mild aggregate formation in keratinocytes harboring the newly identified variants could be observed under normal culturing conditions. This is in line with the mild phenotype observed in the patients and the histological findings reported here. Previously, in vitro studies showed that stress, such as heat or osmotic shock, can result in a more prominent aggregate formation of keratins.^26^, ^27^ However, although we tried inducing stress in KRT10‐construct transfected HaCat cells, the results of stress‐induced aggregate formation for the linker L12 domain variants were inconclusive.

Here, we report for the first time, to our knowledge, that three different missense variants in the L12 linker domain of KRT10 are causal for an atypical, milder form of EI. Similar to other keratins (KRT14, KRT5, KRT1) expressed in the different layers of the epidermis, pathogenic variants in the linker L12 domain are associated with a milder or atypical phenotype (EBS_loc_, EBS_loc_, and EI with PPK, respectively) and are less destructive to the formation of intermediate filaments. Perhaps variants in the linker L12 domain region of KRT10 or other keratins known to be associated with disease may have gone unnoticed because of a clinically barely detectable phenotype.

## CONFLICT OF INTEREST STATEMENT

None declared.

## Supporting information


Figure S1.

Figure S2.

Figure S3.
