# Supplementary material for: Variants in the L12 linker domain of KRT10 are causal to atypical epidermolytic ichthyosis
Source: J Dermatol. 2024 Jul 29;51(9):1180–6. doi: 10.1111/1346-8138.17395 (PMC11484123; doi:10.1111/1346-8138.17395)
Supplement: Supplementary file 1 — Figure S1. [file JDE-51--s002.zip › jde17395-sup-0001-FigureS1.docx]

**Figure S1. Supplementary clinical and histopathological features of the patient of family 1. A**.H&E staining of the patient's (II-1) skin biopsy of family 1, showed eosinophilic spongiosis (asterisks). **B**. Immunofluorescent staining of FLG (green) and nuclei (blue) of a control sample (left panel) and the index patient (right panel) showing separation of the stratum granulosum. **C.** Clinical presentation of the index patient at 2 years of age. At the back of the patient and at his feet blister formation and desquamation can be observed.
