# Supplementary figures and images for: Variants in the L12 linker domain of KRT10 are causal to atypical epidermolytic ichthyosis
Source: J Dermatol. 2024 Jul 29;51(9):1180–6. doi: 10.1111/1346-8138.17395 (PMC11484123; doi:10.1111/1346-8138.17395)

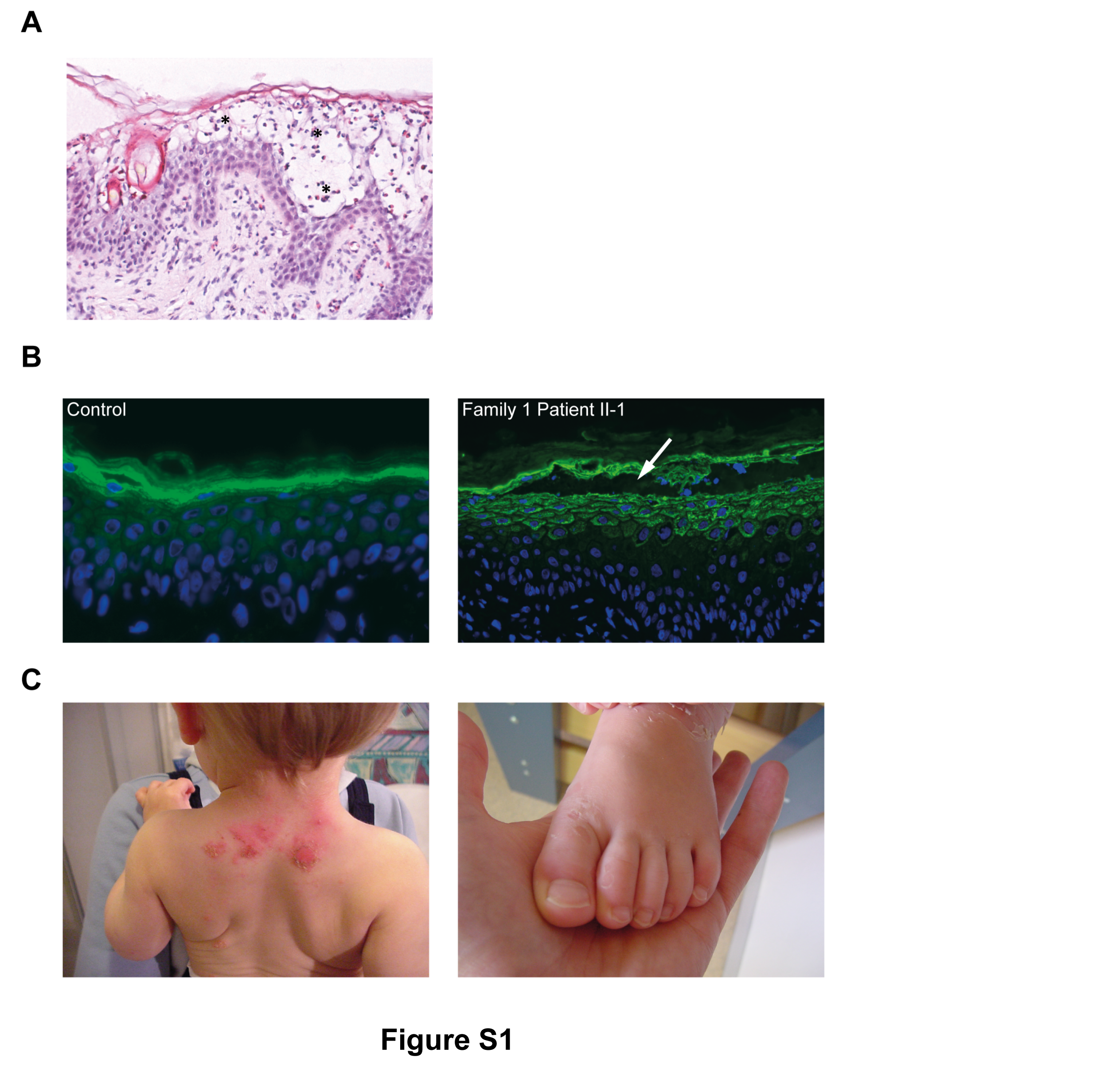

Supplement: Supplementary file 1 — Figure S1. [file JDE-51--s002.zip › jde17395-sup-0001-FigureS1.tif]

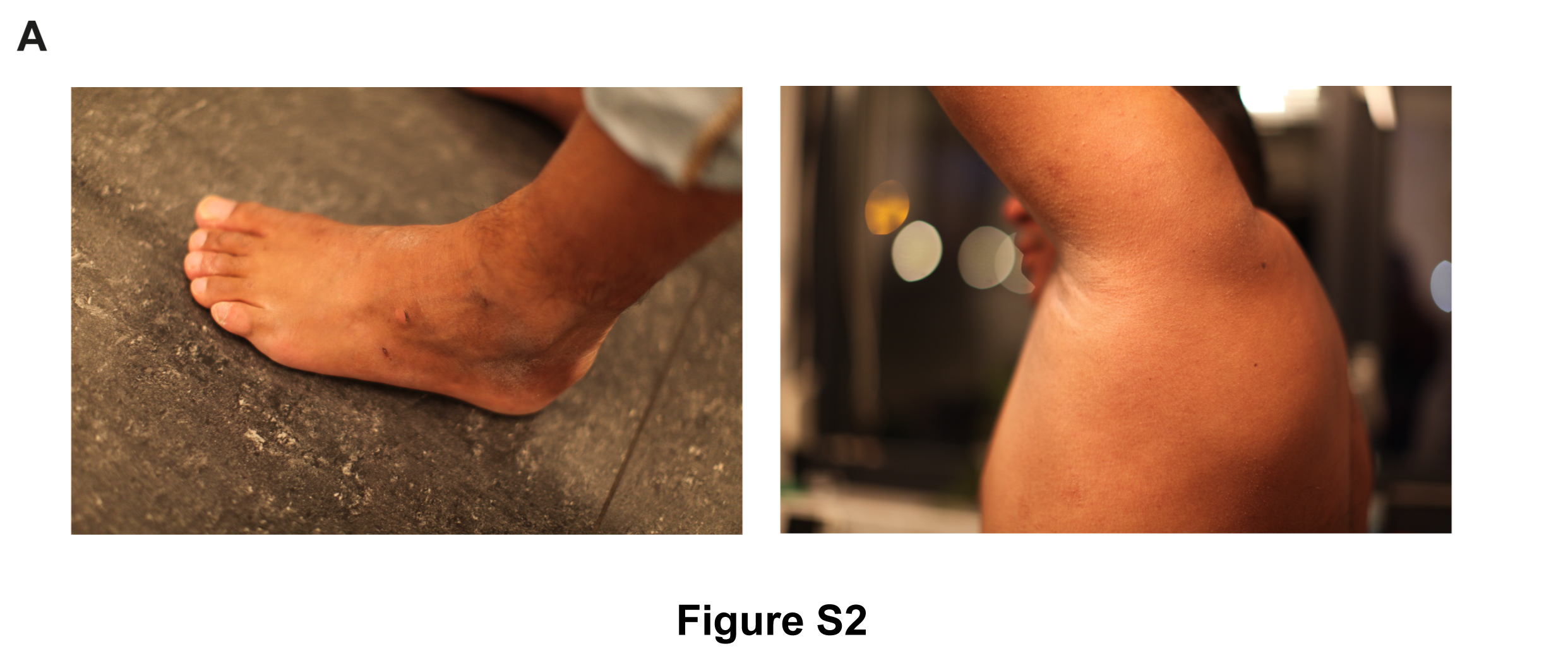

Supplement: Supplementary file 2 — Figure S2. [file JDE-51--s001.zip › jde17395-sup-0002-FigureS2.tif]

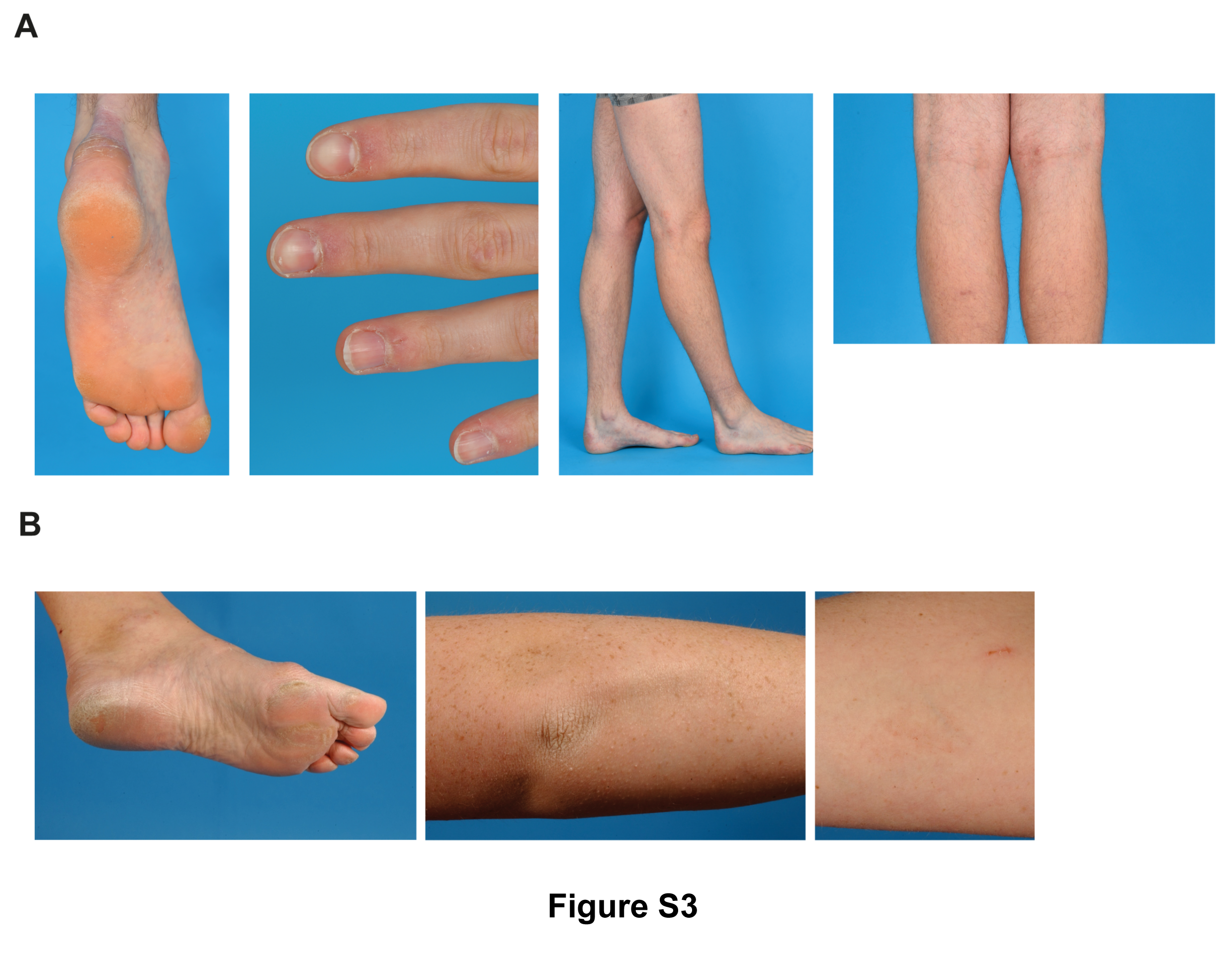

Supplement: Supplementary file 3 — Figure S3. [file JDE-51--s003.zip › jde17395-sup-0003-FigureS3.tif]
