# Supplementary material for: Variants in the L12 linker domain of KRT10 are causal to atypical epidermolytic ichthyosis
Source: J Dermatol. 2024 Jul 29;51(9):1180–6. doi: 10.1111/1346-8138.17395 (PMC11484123; doi:10.1111/1346-8138.17395)
Supplement: Supplementary file 2 — Figure S2. [file JDE-51--s001.zip › jde17395-sup-0002-FigureS2.docx]

Figure S2. **Supplementary clinical and histopathological features in family 2 A****.**. The father (I-1) of the index patient shows superficial erosions and mild ichthyosiform hyperkeratosis on his feet (left panel). On the patient's side, superficial erosions and a mild ichthyosiform hyperkeratosis can be seen (right panel).
