# Supplementary material for: Variants in the L12 linker domain of KRT10 are causal to atypical epidermolytic ichthyosis
Source: J Dermatol. 2024 Jul 29;51(9):1180–6. doi: 10.1111/1346-8138.17395 (PMC11484123; doi:10.1111/1346-8138.17395)
Supplement: Supplementary file 3 — Figure S3. [file JDE-51--s003.zip › jde17395-sup-0003-FigureS3.docx]

**Figure S3. Supplementary clinical features of family 3 A**. Family 3, the index patient (IV-1) as an adult. The patient shows only a very mild plantar hyperkeratosis, subtle collarette desquamation on the fingers and erythematous plaques on the legs. **B**. The mother (III-2) of the index patient shows plantar hyperkeratosis, an area of hyperkeratosis on the elbows and very mild superficial desquamation.
